# Enhancing Agricultural Productivity in Dairy Cow Mastitis Management: Innovations in Non-Antibiotic Treatment Technologies

**DOI:** 10.3390/vetsci12070662

**Published:** 2025-07-12

**Authors:** Lijie Jiang, Qi Li, Huiqing Liao, Hourong Liu, Zhiqiang Wang

**Affiliations:** 1 Department of Customs Inspection and Quarantine, Shanghai Customs University, Shanghai 201204, China; jianglijie@shcc.edu.cn (L.J.); liqi@shcc.edu.cn (Q.L.); liuhourong@shcc.edu.cn (H.L.); 2 College of Veterinary Medicine, Yangzhou University, Yangzhou 225012, China

**Keywords:** mastitis, dairy cows, therapeutic approaches, antibiotic resistance, emerging technologies

## Abstract

Dairy mastitis is a common dairy farming disease. It severely affects the health of dairy cows and the quality and yield of dairy products. This paper reviews the main current mastitis treatments and associated bacterial resistance. It emphasizes the importance of integrated resistance and treatment management. The therapeutic efficacy and resistance associated with commonly used antibiotics such as penicillin, cephalosporins, macrolides and fluoroquinolones are analyzed. The principles, application effects and benefits of non-antibiotic therapies are also discussed, including those of immunotherapy, herbal therapy, probiotic therapy and phage therapy. The paper presents the latest gene editing and nanotechnology advances in the contexts of big data and artificial intelligence. It suggests future research directions such as developing new antibiotics, optimizing treatment and enhancing immunity. In conclusion, effective treatment and management can control dairy cow mastitis. It can guarantee cow health, improve dairy product quality and promote sustainable dairy industry development.

## Simple Summary

Dairy cow mastitis is a widespread disease that harms cow health, reduces milk production, and threatens food safety. Traditional antibiotic treatments are becoming less effective due to growing resistance in bacteria like Staphylococcus aureus and Escherichia coli. This study explores alternative, non-antibiotic approaches to the management of mastitis, aiming to reduce reliance on antibiotics and combat resistance. Researchers reviewed methods such as vaccines, which boost cows’ immunity; herbal remedies, like baicalin and propolis, which fight inflammation and bacteria; probiotics, which support healthy microbes in cows; and phage therapy using viruses that target harmful bacteria. Emerging technologies like gene editing (to make cows more resistant) and nanotechnology (for precise drug delivery) were also examined. These strategies could reduce antibiotic use, slow resistance development, and enhance dairy product safety. By integrating these innovations, the dairy industry can better protect cow health, ensure safer milk, and promote sustainable farming practices, ultimately benefiting both agriculture and public health.

## 1. Introduction

Dairy mastitis is still a major challenge for global health and the global economy, impacting milk production, cow welfare and food safety [1]. Research into antibiotic resistance in mastitis pathogens (*Staphylococcus aureus*, *Escherichia coli*, and *Klebsiella pneumoniae*) has limited the efficacy of conventional treatments [2,3,4]. Antibiotics such as penicillin and cephalosporins are still common, but their overuse has accelerated the dominance of antibiotic-resistant bacteria, leading to a decline in clinical treatment efficacy [5,6,7,8,9].

There is an incomplete understanding of non-antibiotic therapies, such as phage therapy, probiotics and herbal extracts, which show promise but lack long-term safety and standardized protocols for dairy cows. Emerging technologies such as gene editing (e.g., CRISPR) and nanotechnology have not yet been fully integrated into mastitis management. Few studies have evaluated the practical applications or synergy of emerging technologies, like gene editing and nanotechnology, with existing treatments. This highlights the urgent need for non-antibiotic therapies and farm-level interventions to combat resistance.

## 2. Characteristics of Mastitis-Related Pathogens in Dairy Cows

Mastitis in dairy cows is influenced by a combination of factors, including bacterial infections, environmental stressors (e.g., poor hygiene, bedding contamination), management practices (e.g., milking routines, teat care), and systemic immune responses. Among the bacterial pathogens of concern, *Staphylococcus aureus*, *Escherichia coli* and *Klebsiella pneumoniae* are the most prevalent and pose significant challenges to dairy farming [10,11]. Understanding the biology, mechanisms of infection, and prevalence of these pathogens is essential for the prevention and treatment of mastitis. Given the current prevalence of these bacteria and the degrees of drug resistance linked to them, we provide a detailed review of *Staphylococcus aureus*, *Escherichia coli*, *Streptococcus* spp. and *Klebsiella pneumoniae* [12,13,14,15].

### 2.1. Staphylococcus aureus (S. aureus)

*S. aureus*, a Gram-positive bacterium, produces toxins and enzymes, is pathogenic and adaptable and can cause mastitis. *S. aureus* produces a biofilm, protecting itself from the host immune system. It is a common pathogen in mastitis in dairy cows. *S. aureus* produces toxins that destroy cell membranes, causing direct damage to the cow’s lactation system [16,17]. In the early stages of infection, Staphylococcus aureus targets milk cisterns and teats, subsequently invading the mammary duct system. This invasion causes cellular damage, reduces milk production, and leads to ductal blockage by dead cells, ultimately resulting in lactation difficulties and potential milk-production cessation.

*S. aureus,* including the methicillin-resistant strains (MRSA), are resistant to penicillin, and MRSA infections are associated with mastitis cases that are more severe, making effective treatment increasingly difficult [18]. *S. aureus* acquires resistance to β-lactam antibiotics by either producing β-lactamases, which inactivate the drugs, or altering the structure of penicillin-binding proteins (PBPs), which reduces the drugs’ ability to bind to their targets [19].

### 2.2. Streptococcus spp.

*Streptococcus* spp., Gram-positive bacteria, are classified into α-, β-, or γ-types according to their hemolytic properties. All major mastitis-causing bacteria gain initial access to the mammary gland through the teat canal. Once inside, they may ascend into the gland cistern and subsequently spread through the lactiferous ducts, where pathogens such as *streptococci* can proliferate and cause infection. Among them, *Streptococcus uberis*, *Streptococcus agalactiae* and *Streptococcus dysgalactiae* are the most common pathogenic species [20,21,22]. A high percentage of the mastitis in dairy cows, especially in environmental mastitis, is caused by *streptococci*. The primary mastitis-associated species are *Streptococcus uberis* [23] and *Streptococcus dysgalactiae* [24]. The histopathological presentation of *Streptococcus agalactiae* mastitis is distinct, characterized by chronic parenchymal inflammation and fibrosis, reflecting its subclinical nature. In contrast, environmental streptococci such as *Streptococcus uberis* typically induce acute, suppurative lesions with neutrophil infiltration and abscess formation.

*Streptococci* have progressively developed higher rates of resistance to tetracycline, sulfonamides and macrolide antibiotics [25]. *Streptococcus* reduce their antibiotic susceptibility by altering drug targets, decreasing drug intake, or increasing drug efflux.

### 2.3. Escherichia coli (E. coli)

*E. coli*, a Gram-negative bacterium, is found in the environment, and forms part of the normal flora of the intestinal tract. *E. coli* enters the teat canal through contaminated environments or equipment and causes acute mastitis. It is more common in environmental mastitis, especially during hot summer months [13]. Infection leads to clinical mastitis, with a wide range of symptoms. The more severe forms of mastitis can result in tissue damage, cessation of lactation, and even death [26,27]. *E. coli* typically remains localized within the milk ducts of the mammary gland and does not usually invade deeper tissues. Consequently, increasing milking frequency during the early stages of infection can effectively reduce bacterial load and inflammation in mild *E. coli* mastitis. However, this approach is not effective for treating moderate to severe cases, which often require additional interventions. *E. coli* has developed resistance to many antibiotics, especially third-generation cephalosporins. In *E. coli*, the highest proportions of resistance were observed for amoxicillin (28.1%) and tetracycline (23.1%). Resistance to third-generation cephalosporins in *E. coli* from dairy cattle was almost nil in 2006, but reached 2.4% in December 2016. This increase is particularly concerning because these antibiotics constitute one of the latest therapeutic alternatives developed to fight severe infectious diseases in humans [28]. Cefquinome is the fourth generation of cephalosporin, and is approved solely for animal usage; Xia Xiao’s research optimized the current dosage of cefuroxime using the PK/PD model, which can effectively reduce the development of drug resistance [29]. *E. coli* accomplishes this by reducing permeability and efficacy, mainly by producing β-lactamases, altering outer membrane pore proteins, or increasing the active-efflux pumps.

### 2.4. Klebsiella pneumoniae (K. pneumoniae)

This species is Gram-negative, is capable of producing polysaccharide capsules, and demonstrates strong adhesion and anti-phagocytosis ability. *K. pneumoniae* enters the bovine mammary gland primarily through the teat canal, often via contaminated bedding, soil or milking equipment. It causes acute mastitis characterized by severe udder swelling, high fever and systemic illness, with symptoms that may appear similar to those of *E. coli* [12]. In recent years, the detection rate of *K. pneumoniae* in dairy cow mastitis has increased, particularly on farms characterized by lower levels of management. In recent years, national and international epidemiological surveys have shown an increasing trend of *K. pneumoniae* infections in dairy farms [30]. A study by Saddam et al. showed that the bacterial identification of 700 mastitis milk samples from a dairy farm in the Peshawar region of Pakistan revealed that *K. pneumoniae* accounted for 25.7% of the samples [31]. Xiangyun Wu’s study reported that 887 test samples of bedding, feed, feces and milk from five dairy farms in Hubei Province, China, were subjected to isolation and identification, and *K. pneumoniae* was isolated from 26.94% of the samples [32]. The results of the study of Cheng J et al. showed that an epidemiological survey of the incidence of *K. pneumoniae*-infected mastitis on two large-scale farms in China found that the detection rates of *K. pneumoniae* in mastitis milk samples were 53% and 62%, respectively, and that the above data indicated that *K. pneumoniae* is gradually deepening its impact on dairy farms [33].

*K. pneumoniae* is particularly multi-drug resistant, with an increasing rate of resistance to carbapenem antibiotics. *K. pneumoniae* reduces the antimicrobial efficacy of drugs mainly through the production of ultrabroad-spectrum β-lactamases (ESBLs) or carbapenemases (KPCs), as well as by altering the permeability of the outer membrane and increasing the activity of its efflux pumps.

### 2.5. Update on Drug Resistance

The growing problem of the resistance of dairy mastitis pathogens to commonly used antibiotics poses a significant challenge for clinical management. In recent years, the phenomenon of multidrug resistance in *K. pneumoniae* has attracted widespread attention [30,34]. In particular, carbapenemase-positive *K. pneumoniae* is highly resistant to almost all β-lactam antibiotics, which makes the clinical treatment options extremely limited. In addition, in some regions, the detection rate of *K. pneumoniae* has increased dramatically, further exacerbating the severity of the resistance problem.

In addition to *K. pneumoniae*, common mastitis-associated pathogens in dairy cows also exhibit widespread resistance. *E. coli* has shown increased resistance to third-generation cephalosporins, and multi-drug resistant (MDR) strains are becoming more common [35]. Methicillin-resistant *S. aureus* (MRSA) is a major problem and is driven by the methicillin-resistant gene (*mecA*), which encodes an altered penicillin-binding protein (PBP) that reduces antibiotic binding [36]. *Streptococcus* spp. have increased resistance to tetracyclines and macrolides. The antimicrobial susceptibility results described by Dhital showed that *Streptococcus* spp. were resistant to tetracycline (86.30%), neomycin (79.45%) and oxacillin (73.97%) [37]. Regarding the evaluation of the varying levels of resistance in antimicrobial classes in Martins’ study, the highest frequency was observed for macrolides [23].

## 3. Non-Antibiotic Applications

With the growing problem of antibiotic resistance, non-antibiotic treatments are gaining attention. Here are some of the emerging treatments, their rationales, their means of efficacy and their potential benefits:

### 3.1. Vaccines

Vaccines (*S. aureus*, StartVac; *E. coli*, *E. coli* vaccine) targeting pathogens associated with mastitis aim to enhance the immune response in dairy cows, reduce antibiotic use, and improve disease resistance (Table 1). Vaccines targeting *S. aureus* and *E. coli* consistently increased IgG levels in serum and milk, indicating enhanced immune activation [38,39]. Schukken and Pipers reported that vaccinated herds had reduced incidence of new *S. aureus* and coagulase-negative Staphylococcus infections, as well as shorter disease durations [40]. Frick observed no statistically significant reduction in the incidence or duration of clinical mastitis in vaccinated dairy cows, despite an increase in milk production [41]. In summary, most vaccines have limited efficacy against environmental pathogens such as *streptococci*, highlighting the narrow scope of the protective effects of these vaccines. In addition to the two vaccines mentioned above, the *S. uberis* vaccine has also been used. The *S. uberis* vaccine can inhibit the adhesion and internalization of *S. uberis* to breast epithelial cells in vitro [42].

The inconsistency in results stems from multiple factors. Variability in vaccine design—such as antigen composition (e.g., recombinant proteins vs. inactivated strains)—alters immunogenicity. Methodological limitations, including small sample sizes, short follow-up periods and failure to account for farm-specific practices (e.g., hygiene, pathogen prevalence) further complicate comparisons. Biological factors, such as individual cows’ immune status (e.g., age, pregnancy stage), also influence responsiveness.

While multivalent vaccines targeting *S. aureus* and *E. coli* are already commercially available, the current vaccines remain limited in their coverage of the diverse mastitis-pathogen spectrum. To bridge this gap, future research should prioritize the development of vaccines against underrepresented pathogens such as *S. dysgalactiae*, and environmental Gram-negative organisms like *K. pneumoniae*, which are increasingly implicated in chronic and recurrent infections. Mucosal immunization approaches, such as intramammary delivery, could enhance local udder immunity, while genetic editing might optimize antigens to disrupt pathogen colonization (e.g., by targeting biofilm-forming proteins). Large-scale, multi-center trials with long-term follow-up are critical in validating efficacy across diverse settings. Ultimately, vaccines should be integrated with non-antibiotic therapies (e.g., probiotics, phage therapy) to address the limitations of the vaccine approach and achieve comprehensive mastitis control.

### 3.2. Herbal Treatment

Scutellaria baicalensis, a traditional herb with antibacterial, anti-inflammatory and immunomodulatory properties, has shown good antibacterial effects in some studies, along with fewer side effects [46,47,48]. It is natural and safe and can reduce drug residues. Previous research indicates that baicalin primarily inhibits bacteria by inhibiting biofilm formation, disrupting suilysin’s secondary structure, inducing autophagy, and inhibiting penicillin-binding proteins [49,50,51,52]. Baicalin can enhance the cytotoxicity of neutrophils against *S. aureus* and effectively inhibit the inflammatory responses of mammary epithelial cells in cows. Relevant studies have shown that various herbal extracts, such as lemon balm and peppermint oil, not only have good antibacterial effects, but also have significant anti-inflammatory activities [53,54,55,56]. These studies have cited reported preliminary in vitro findings demonstrating reduced bacterial load in mammary cell cultures treated with this therapy. But these results do not confirm in vivo efficacy. To establish therapeutic relevance, future research must prioritize multicenter, double-blind, randomized controlled trials in live animals under field conditions, as such studies are the standard for evaluating interventions in veterinary medicine.

Propolis has been found to be a good alternative to conventional antimicrobial drugs used in clinical and subclinical mastitis, and studies have been conducted to validate this in in vivo and in vitro models, in animals such as rats, goats and dairy cows. The results of the study by Bacic et al. showed in vitro antimicrobial activity against common mastitis pathogens, using propolis extracts, and clinical trials were conducted in Holstein cows [57]. The results demonstrated that a one percent intramammary propolis preparation had high antimicrobial and antioxidant activities both in vitro and in vivo. The results of Wang et al. showed that when using the mammary epithelial cells of dairy cows as the object of study, and using a variety of mastitis-pathogenic bacteria to induce the establishment of cellular inflammatory injury model, it was found that 15 μg/mL propolis had a better protective effect on the mammary epithelial cell injury induced by the mastitis-pathogenic microorganisms associated with dairy cows [58].

### 3.3. Phage Therapy

Phages are highly specific for a particular pathogen and can infect and kill that pathogen. Some studies have shown that phages have good antibacterial activity and are not prone to drug resistance. Gill treated 24 Holstein cows with subclinical mastitis (caused by *S. aureus*) with phage therapy. The treatment consisted of an intramammary injection of 10 mL of 1.25 × 10^11^ PFU/mL phage K or saline administered once daily for 5 days [59]. It was found that the cure rate in the phage-treated group was 16.7%, whereas none in the saline group were cured; it was also found that the phage survived in milk for up to 36 h. Kwiatek’s findings reveal the isolation of a novel lysogenic phage (MSA6) from the mastitis-causing agent in dairy cows. This phage can infect various bovine- and human-derived *S. aureus* strains, indicating its potential as a versatile, broad-spectrum alternative to the antibiotics used against *S. aureus* [60]. Dias’s results showed that pathogenic *S. aureus* was isolated from cows suffering from mastitis and screened for phages, and 10 phage strains were isolated [61]. These phages possessed the key features of phage therapy, i.e., broad host spectrum, high catalytic activity and high stability. Fenton et al.’s study showed that phage-derived peptidase (CHAPK) was used to rapidly interrupt the formation of *S. aureus* biofilm; it was found that, within 4 h, purified CHAPK could completely eliminate the biofilm formed by *S. aureus*. At the same time, CHAPK was able to efficiently block the biofilm formation [62].

CRISPR-Cas systems have enabled strategies such as Phage-Delivered Resistance Eradication with Subsequent Antibiotic Treatment (PRESA), which combines phage-mediated gene editing with targeted antibiotic use. In PRESA, phages are engineered to carry CRISPR-Cas systems that specifically cleave antibiotic resistance genes (e.g., mecA in MRSA, or blaCTX-M in *E. coli*), rendering pathogens susceptible to conventional therapies. By eliminating resistance genes rather than relying solely on phage lysis, PRESA reduces the risk of resistance recurrence and allows for a more judicious use of antibiotics. However, challenges such as delivery efficiency, off-target effects and regulatory hurdles must be addressed to realize its full potential in mastitis treatment. Phage-delivered CRISPR targeting efficiently eradicated and blocked the transfer of the antibiotic resistance plasmas in Liu et al.’s research. PRESA decreased the bacterial load by over six logs and five logs in vitro and in vivo, respectively. Importantly, while lytic phages induced mutational phage resistance at 24 h in vitro and 48 h in vivo, PRESA demonstrated a constant effect and revealed no resistant mutants. Genes involved in DNA mismatch repair were upregulated in cells undergoing Cas9-based plasmid cleavage, which may reduce the development of mutations [63].

### 3.4. Probiotics

Probiotics are defined as “live microorganisms that, when administered in adequate amounts, confer a health benefit on the host.” These microorganisms have the capacity to promote the balanced development of the micro-ecology in the host’s body by colonizing the intestinal tract, reproductive system and other bodily organs. This colonization is beneficial to the body’s health and results in a certain level of activity [64]. Studies have shown that probiotics can penetrate the small-intestinal epithelium via endocytosis through the dendritic cells of the intestinal epithelium and reach the mammary tissue site via the internal circulation [65]. It is important to note that the study referenced was conducted using lactobacilli isolated from human milk, and the reported immunomodulatory effects were observed in human subjects. The potential application of these findings to bovine mammary tissue remains speculative and is based on an extrapolation from small-intestinal epithelial responses—an assumption that the authors themselves acknowledge to be a subject of debate. Therefore, while promising, such approaches require rigorous validation in animal models before they can be considered relevant to mastitis prevention in dairy cattle. In Kitching et al.’s study, the authors describe the optimization, for the first time, of a formulation of Lactococcus lactis DPC3147 that produces the two-component bacteriocin lacticin 3147, in a liquid paraffin-based emulsion (a formulation subsequently designated ‘live bio-therapeutic’), and compare it to the commercial antibiotic formulation TerrexineTM, with a view to the treatment of cows with clinical/sub-clinical mastitis. The results showed that the live bio-therapeutic formulation displayed a 47% cure rate, compared to a 50% cure rate for a commercial antibiotic control, with respect to the curing of cows with clinical/sub-clinical mastitis. The study suggests that a larger field trial to further demonstrate efficacy is warranted [66]. Soleimani’s study investigated the antagonistic activities of *Lactobacillus acidophilus* DSM 20079, *Lactobacillus plantarum* ATCC 8014, *Lactobacillus casei* ATCC 39392, and *Lactobacillus reuteri* ATCC 23272 against *S. aureus* isolates from bovine mastitis and the reference strain *S. aureus* ATCC 25923. And the results showed that Lactobacillus plantarum and its antimicrobial compounds have the strongest bacteriostatic effect and can be considered an option for the control of *S. aureus* [67].

The treatment of mastitis in dairy cows has changed from a single antibiotic treatment to the combined use of several therapeutic methods. The continued emergence of non-antibiotic and novel treatments has provided new ideas and tools for the prevention and treatment of mastitis in dairy cows. Future studies should continue to investigate the safety and efficacy of these methods, with the aim of providing a more comprehensive and effective solution for the control of mastitis in dairy cows.

## 4. Emerging Technologies for Next-Generation Therapies

### 4.1. Modification of Cow Mammary Cells by Gene Editing Technology

Gene editing technology, which allows the precise replacement of target genes, is one of the currently popular therapeutic approaches to the treatment of hereditary diseases. Specific genes in dairy cow mammary cells are modified using gene editing techniques such as CRISPR-Cas to increase their resistance to pathogens and reduce pathogen attack. It is also possible to edit genes associated with antiviral or antimicrobial properties to enable dairy cow mammary cells to resist specific pathogens more effectively. It has been shown that gene editing techniques can successfully increase the resistance of animals to certain diseases. For example, by editing the TLR4 gene in cows, their resistance to *E. coli* can be increased. Gene-edited increased resistance is heritable, and can provide long-term protective effects. By increasing the cow’s own resistance, this technique reduces dependence on external antibiotics and reduces the risk of resistance. Shandilya et al.’s findings suggest that TLR4 is essential for eliciting inflammation in response to LPS; however, the exacerbated gene and protein expression in TLR4 KO cells in response to a *Mycobacterium avium* ssp. paratuberculosis cell lysate suggests a different mechanism of infection and host response for *Mycobacterium avium* ssp. paratuberculosis, at least in terms of how it interacts with TLR4. These novel findings show various potential divergent roles for TLR4 in mycobacterial infections, and this may have important consequences for the therapeutic control of inflammation in cattle [68]. It is important to note that the referenced study specifically investigated *Mycobacterium paratuberculosis*, and its outcomes cannot be generalized to all *Mycobacteria* or all mastitis-associated pathogens. While the study demonstrated promising immunomodulatory effects and protective mechanisms in a model of chronic mycobacterial infection, applying these findings to mastitis pathogens would require further investigation. Deb et al.’s findings show that targeting the *E. coli* CTX-M gene using a CRISPR/cas9 cassette reveals changes in the cefotaxime antibiotic phenotype [69]. Liu used the pdCas9-C-Tet1-SgRNA 2.0 system to regulate the methylation status of the AKT1 promoter; the degree of methylation of the AKT1 promoter was significantly reduced in BMMECs, while AKT1 protein levels increased. These results indicate that demethylation guided by the pdCas9-C-Tet1-SgRNA 2.0 system on the AKT1 promoter can reactivate the expression of AKT1 and AKT1/mTOR signaling pathway-related proteins by reducing the AKT1 promoter’s methylation level and promoting the recovery milk protein expression in BMMECs, thereby alleviating the symptoms of mastitis [70]. Shandilya et al. demonstrate that TLR4 knockout via CRISPR in *Mycobacterium*-infected mammary epithelial cells modulates inflammatory responses, suggesting a therapeutic strategy to mitigate excessive inflammation in cattle mastitis. This aligns with Deb et al.’s approach, in which CRISPR/Cas9 targeting of the *E. coli* CTX-M gene—a key contributor to antibiotic resistance—reverses cefotaxime resistance, offering a potential avenue to combat multidrug-resistant pathogens in livestock. Notably, Liu et al. introduce an epigenetic dimension by employing the pdCas9-C-Tet1-SgRNA 2.0 system to demethylate the AKT1 promoter in bovine mammary epithelial cells (BMMECs). This intervention not only reactivates AKT1 expression and its downstream mTOR pathway, but also restores milk protein synthesis, directly alleviating mastitis symptoms. Collectively, these studies underscore the transformative potential of CRISPR tools beyond conventional gene editing. While Shandilya and Deb focus on genetic disruption to modulate immunity and antibiotic efficacy, Liu’s work expands the toolkit to epigenetic regulation, demonstrating that reversible methylation changes can rescue pathological phenotypes. The convergence of these approaches—targeting immune receptors, antibiotic resistance genes and epigenetic modifiers—highlights a multi-pronged strategy for managing complex diseases like mastitis, which involve immune dysregulation, microbial pathogenesis and cellular dysfunction.

### 4.2. Nanotechnology and Drug-Delivery Systems

Typically, ligands specific to the cognate receptors expressed on the intended target cell type are conjugated to the nanoparticle’s surface. This approach, often called ‘active targeting’, seems to imply that the conjugated ligand imbues the nanoparticle with a homing capacity. Using nanoparticles as a carrier aims to deliver the drug precisely to the lesion site for a targeted and slow release of the drug. This requires the design of nanoparticles with specific targeting molecules, so that they specifically recognize and bind to mastitis lesions, reducing the damage to normal tissue. By controlling the release rate of the nanoparticles, a sustained release of the drug is achieved, prolonging the therapeutic effect. Nanodrug-delivery systems have shown promise in the treatment of a wide range of diseases, including cancer and cardiovascular disease [71,72], along with reduced drug damage to normal tissue and fewer side effects. Wierzbicki et al.’s study showed that all tested nanoparticles, except iron and platinum, had biocidal properties against biological isolates, with silver–copper complexes being the most effective. In addition, the nanoparticles showed synergistic effects, while low concentrations of nanoparticles had no toxic effects on BME-UV1 and HMEC cells [73]. Wang et al.’s research developed an intramammary (IMA) drug-delivery system (IMDS) for a lasalocid solid dispersion for the treatment of bovine mastitis. The IMDS provides effective treatment, with higher clinical and microbiological cure rates for mastitis, and an acceptable safety profile for treated cows [74]. Hashem et al.’s study showed that in vitro and in vivo results demonstrated the antioxidant and anti-inflammatory efficacy of berberine chloride/CH-NPs and cyperus rotundus rhizomes/CH-NPs (BER/CH-NPs and CRE/CH-NPs) in low doses, with minimal damage to the liver and kidney functions, positing the promising uses of these compounds in mastitis treatment [75]. Yadav found that ALA-NS had a higher zone of inhibition and lower minimum inhibitory concentration (MIC) against mastitis-causing pathogens in vitro, compared to ALA and ceftazidime alone. ALA-NS restored changes in oxidative biomarkers (superoxide dismutase, catalase, glutathione, TBARs and protein carbonyls) and histopathological changes in lipopolysaccharide (LPS)-treated rats. An alpha-linolenic acid-based nanosuspension (ALA-NS) possessed in vitro antimicrobial activity and protected against LPS-induced mastitis in rats [76].

**Figure 1:**
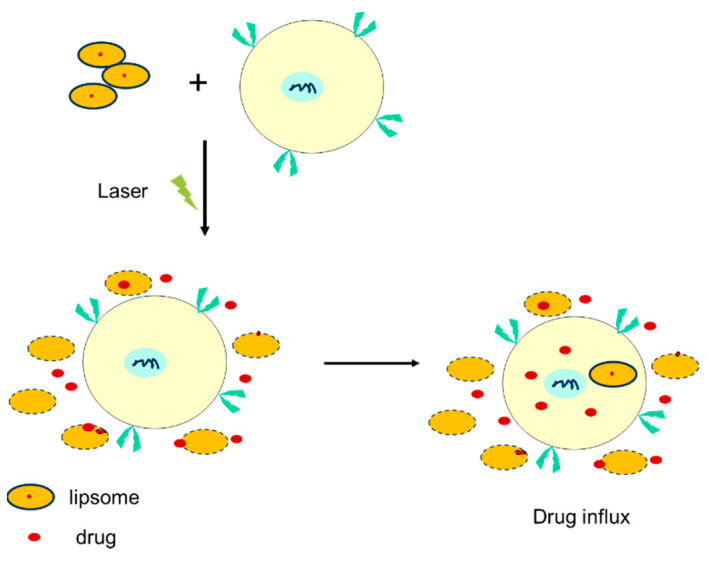
Light treatment of drug-loaded liposomes results in the release of the drug.

A new drug-delivery strategy based on a nano material has been developed: a ‘SMART nanoparticle’ could be used in bovine mastitis therapy. Smart nanotechnology platforms, such as liposomes, deliver drugs by releasing them in response to internal stimuli (e.g., pH, reducing agents, or specific enzymes) or external stimuli (e.g., light, magnetic fields, or ultrasound), thereby enhancing the targeting and precision of the drug delivery [77,78]. Wang and Kim modified the block co-polymer Pluronic F127 by attaching cinnamoyl groups (CF127) and immobilized it on the surface of EPC liposomes, resulting in a triggered release of its water-soluble payload in response to a temperature change [79]. Yavlovich prepared photo-triggerable liposomes from photo-polymerizable diacetylene phospholipid and dipalmitoyl phosphatidylcholine, which released calcein (a fluorescent dye entrapped in liposome) upon treatment with UV light (254 nm) (Figure 1) [80]. This is particularly important in bovine mastitis, for which the conventional therapies result in high toxicity and poor specificity and induce multi-drug resistance [81,82]. Smart nanocarriers can be engineered to release their payloads in response to an internal stimulus that is specific to certain microenvironments, such as pH, ions, hypoxia, enzymes, or proteins. Furthermore, dual- or multi-responsive smart nanocarriers have been developed to further ensure the targeted release.

### 4.3. Big Data and Artificial Intelligence (AI)

Using big data and artificial intelligence (AI) technology, this approach collects and analyzes cow health data and builds a predictive model to provide early warning and the precise treatment of mastitis. An early warning system can be established by monitoring the physiological indicators and environmental parameters of cows to detect early signs of mastitis in time. And, according to the specific conditions of individual cows, personalized treatment plans can be formulated for precise treatment. Some research institutes have started to develop dairy cow health-management platforms using big data and AI technologies that enable the real-time monitoring and intelligent diagnosis of dairy cow health. Automated and intelligent management systems can also be used to improve the efficiency and management of dairy farming and reduce the incidence of mastitis.

The future treatment of mastitis in dairy cows will increasingly rely on cutting-edge technologies such as gene editing and biotechnology, nanotechnology, and big data and AI. The development of these technologies will not only improve therapeutic efficacy and reduce the use of antibiotics, but also improve the health of dairy cows and the safety of dairy products. Through the comprehensive application of these new technologies, it is expected that precise prevention and treatment of mastitis in dairy cows will be achieved, and the sustainable development of the dairy industry will be promoted.

## 5. The Balance Between Treatment Efficacy and Resistance

The utilization of antibiotics for extended durations and at elevated dosages can precipitate an escalation in bacterial resistance, thereby engendering treatment inefficacy. The emergence of drug-resistant bacteria not only makes treatment more difficult, but also poses the risk of these species being transmitted to humans through the food chain, threatening public health. In the future, the treatment of mastitis will be strictly rationalized, in order to avoid the unnecessary use of antibiotics and to ensure the necessity and rationality of the drug use. At the same time, different types of antibiotics will be regularly changed to reduce the overuse of a single antibiotic and slow down the development of drug resistance. A bacterial resistance monitoring system will be established to regularly detect changes in pathogen resistance and adjust treatment programs in a timely manner.

Non-antibiotic treatments do not usually lead to an increase in bacterial resistance, but in some cases their therapeutic effects may not be as strong as those of antibiotics. Combining antibiotic and non-antibiotic treatments to take advantage of their respective strengths improves the overall effectiveness of treatment. At the same time, vaccines and other methods can be used to boost the cow’s own immunity and reduce reliance on drugs. Improvements in the feeding environment and management methods are critical for reducing the incidence of mastitis in dairy cows [83,84,85].

## 6. Discussion

Antibiotics remain the most effective treatment for acute bacterial infections, such as those caused by *S. aureus* or *E. coli*, achieving high cure rates in severe cases. However, their overuse, combined with inappropriate use patterns such as incorrect dosing (e.g., subtherapeutic or excessive doses), prolonged treatment durations beyond clinical necessity, and reliance on single-antibiotic regimens without rotation, significantly contributes to the development of antibiotic resistance. These practices exert selective pressure on bacterial populations, favoring the survival and proliferation of resistant strains. Additionally, misuse in agricultural settings, such as administering antibiotics for growth promotion or prophylaxis without proper veterinary oversight, further exacerbates resistance emergence. Probiotics, such as *Lactobacillus* strains, demonstrate moderate efficacy in reducing infection recurrence by inhibiting pathogen colonization. However, their reliability is compromised by strain variability and poor standardization, rendering their herd-level application results unreliable despite their low cost and safety. Phage therapy offers high specificity and avoids resistance by targeting pathogens like *S. aureus* biofilms. Nevertheless, its narrow spectrum necessitates pathogen-tailored phage libraries, which are costly to develop, and the approach faces regulatory ambiguity (e.g., unclear classification as a drug or biologic), complicating commercialization. Herbal extracts, such as baicalein or propolis, provide anti-inflammatory and antimicrobial effects but suffer from inconsistent potency due to batch variability and lack of quality control, compounded by long treatment durations and unclear long-term safety. Nanotechnology enhances drug-delivery precision, but poses risks of toxicity, high production costs and unresolved environmental concerns from nanoparticle residues, given the lack of global safety standards for milk. Gene editing (e.g., CRISPR) holds transformative potential by targeting pathogen receptors in order to boost immunity. However, technical hurdles such as editing efficiency in bovine cells, ethical dilemmas and stringent regulations render it economically unviable for most farms due to its exorbitant costs. Advancements in artificial intelligence (AI) and the utilization of big data through predictive analytics have enhanced the capacity for early detection. However, the implementation of these technologies necessitates the establishment of costly infrastructure and the acquisition of high-quality data inputs, a process that excludes small-scale producers and is susceptible to algorithmic biases in heterogeneous environments.

Non-antibiotic therapies confront substantial systemic barriers to practical application. The development of phage applications and nanotechnology necessitate multimillion-dollar investments and specialized infrastructure. Gene editing’s high costs and regulatory restrictions limit accessibility. Management challenges include inconsistent administration of probiotics and herbal extracts, the need for real-time pathogen identification for phage therapy and AI’s reliance on standardized data in diverse settings. Regulatory gaps, including the ambiguity of policies surrounding nanoparticle residues and phage classification, further impede the adoption of these approaches. Small-scale farms encounter significant challenges in implementing these technologies due to the large technical and financial demands, compounding equity issues. Non-antibiotic approaches often necessitate centralized laboratories or industrial partnerships, leaving marginalized producers dependent on costly external resources. Addressing these interconnected challenges, including cost, accessibility, scalability and regulatory clarity, is imperative to realize the full potential of antibiotic alternatives. Without this, these alternatives remain confined to theoretical applications, failing to meet the diverse and critical needs of dairy farming systems around the world, in which simplicity, affordability and reliability are paramount.

## 7. Conclusions

Dairy mastitis, a disease prevalent in global dairy farming, negatively impacts cow health and the quality of dairy products, resulting in economic losses. This review assesses treatments while addressing knowledge gaps, particularly the concern regarding antibiotic resistance in pathogens like *S. aureus*, *E. coli* and *K. pneumoniae*. Penicillin, cephalosporins, macrolides and fluoroquinolones remain widely used, but face challenges due to resistance mechanisms. *K. pneumoniae* is increasingly prevalent on dairy farms and exhibits multidrug resistance, including carbapenem resistance via enzymes like KPC. This underscores the need for alternatives.

Non-antibiotic approaches like vaccines, herbal extracts, probiotics and phage therapy have shown promise. Vaccines, despite inconsistent efficacy in clinical trials, have been shown to reduce infection severity and boost immunity (e.g., *S. aureus* vaccines increase IgG levels). Herbal therapies, such as baicalein and propolis, have demonstrated antibacterial and anti-inflammatory properties, inhibiting biofilms and reducing inflammation. Probiotics, such as Lactobacillus strains, modulate the microbiota of the udder, inhibiting pathogens. Phage therapy uses enzymes such as CHAPK to target *S. aureus* biofilms. Advanced technologies like CRISPR-mediated gene editing (e.g., AKT1 expression for improved milk protein synthesis) and nanotechnology (e.g., silver–copper nanoparticles for targeted drug delivery) have created innovative tools. The integration of big data and artificial intelligence facilitates both the early detection of pathogens and personalized treatments, enhancing management strategies.

There are several issues that require attention, despite these advances. Non-antibiotic therapies need standardized protocols and large-scale validation. The epidemiology and resistance mechanisms of *K. pneumoniae* require further study. Farm-level interventions referencing antibiotic stewardship have been characterized by ineffective implementation. The research must prioritize mechanistic studies on the efficacy of phage-based and herbal approaches, interdisciplinary technologies and policies designed to curb resistance. By integrating antibiotic stewardship, non-antibiotic strategies, and tools, we can better control mastitis, ensuring cow health, a safe dairy industry and a sustainable dairy industry. The transition to integrated, resistance-aware strategies is imperative in order to address current and future pathogens.

## Figures and Tables

**Table 1:** Target pathogenic bacteria, usage methods, efficacy/recommendations, and monitoring indicators associated with vaccines.

	Outcome	Target	Effect Size	Reference
1	Increased IgG	*S. aureus*, *E. coli*	Moderate to significant	Leitner [43] Wilson [39].
2	Reduced mastitis severity	*S. aureus*, *E. coli*	Modest to significant	Schukken [40] Chang [44]
3	Improved milk-yield	*E. coli*	Variable; not always statistically significant	Bradley [45] Freick [41]
4	Limited efficacy	Environmental pathogens	Inconsistent or null	Landin [38] Freick [41]

## Data Availability

The original contributions presented in this study are included in the article. Further inquiries can be directed to the corresponding authors.

## References

[1] Olde Riekerink R.G., Barkema H.W., Kelton D.F., Scholl D.T. (2008). Incidence rate of clinical mastitis on Canadian dairy farms. J. Dairy Sci..

[2] Krömker V., Leimbach S. (2017). Mastitis treatment-Reduction in antibiotic usage in dairy cows. Reprod. Domest. Anim..

[3] Liang D., Arnold L.M., Stowe C.J., Harmon R.J., Bewley J.M. (2017). Estimating US dairy clinical disease costs with a stochastic simulation model. J. Dairy Sci..

[4] Rollin E., Dhuyvetter K.C., Overton M.W. (2015). The cost of clinical mastitis in the first 30 days of lactation: An economic modeling tool. Prev. Vet. Med..

[5] Hu X., Zhang N., Fu Y. (2019). Role of Liver X Receptor in Mastitis Therapy and Regulation of Milk Fat Synthesis. J. Mammary Gland. Biol. Neoplasia.

[6] Hu H., Fang Z., Mu T., Wang Z., Ma Y., Ma Y. (2021). Application of Metabolomics in Diagnosis of Cow Mastitis: A Review. Front. Vet. Sci..

[7] Garcia S.N., Mpatswenumugabo J.P.M., Ntampaka P., Nandi S., Cullor J.S. (2023). A one health framework to advance food safety and security: An on-farm case study in the Rwandan dairy sector. One Health.

[8] Zhylkaidar A., Oryntaev K., Altenov A., Kylpybai E., Chayxmet E. (2021). Prevention of Bovine Mastitis through Vaccination. Arch. Razi Inst..

[9] Campos B., Pickering A.C., Rocha L.S., Aguilar A.P., Fabres-Klein M.H., de Oliveira Mendes T.A., Fitzgerald J.R., de Oliveira Barros Ribon A. (2022). Diversity and pathogenesis of Staphylococcus aureus from bovine mastitis: Current understanding and future perspectives. BMC Vet. Res..

[10] Duse A., Persson-Waller K., Pedersen K. (2021). Microbial Aetiology, Antibiotic Susceptibility and Pathogen-Specific Risk Factors for Udder Pathogens from Clinical Mastitis in Dairy Cows. Animals.

[11] Heikkilä A.M., Liski E., Pyörälä S., Taponen S. (2018). Pathogen-specific production losses in bovine mastitis. J. Dairy Sci..

[12] Wusiman M., Zuo J., Yu Y., Lv Z., Wang M., Nie L., Zhang X., Wu J., Wu Z., Jiang W. (2024). Molecular characterization of Klebsiella pneumoniae in clinical bovine mastitis in 14 provinces in China. Vet. Res. Commun..

[13] Goulart D.B., Mellata M. (2022). *Escherichia coli* Mastitis in Dairy Cattle: Etiology, Diagnosis, and Treatment Challenges. Front. Microbiol..

[14] Pérez V.K.C., Costa G.M.D., Guimarães A.S., Heinemann M.B., Lage A.P., Dorneles E.M.S. (2020). Relationship between virulence factors and antimicrobial resistance in Staphylococcus aureus from bovine mastitis. J. Glob. Antimicrob. Resist..

[15] Kabelitz T., Aubry E., van Vorst K., Amon T., Fulde M. (2021). The Role of *Streptococcus* spp. in Bovine Mastitis. Microorganisms.

[16] Rainard P., Foucras G., Fitzgerald J.R., Watts J.L., Koop G., Middleton J.R. (2018). Knowledge gaps and research priorities in Staphylococcus aureus mastitis control. Transbound. Emerg. Dis..

[17] Zaatout N., Ayachi A., Kecha M. (2020). Staphylococcus aureus persistence properties associated with bovine mastitis and alternative therapeutic modalities. J. Appl. Microbiol..

[18] Kløve D.C., Jensen V.F., Astrup L.B. (2022). First Finding of a Methicillin-Resistant Staphylococcus aureus (MRSA) t304/ST6 from Bovine Clinical Mastitis. Antibiotics.

[19] Singh I., Roshan M., Vats A., Behera M., Gautam D., Rajput S., Rana C., De S. (2023). Evaluation of Virulence, Antimicrobial Resistance and Biofilm Forming Potential of Methicillin-Resistant Staphylococcus aureus (MRSA) Isolates from Bovine Suspected with Mastitis. Curr. Microbiol..

[20] Bechtold V., Petzl W., Huber-Schlenstedt R., Gangl A., Sorge U.S. (2024). Antimicrobial resistance of Streptococcus dysgalactiae, Streptococcus agalactiae, and Streptococcus canis in quarter milk samples from Bavaria, Southern Germany, between 2012 and 2022. J. Dairy Sci..

[21] Miotti C., Cicotello J., Suarez Archilla G., Neder V., Alvarado Lucero W., Calvinho L., Signorini M., Camussone C., Zbrun M.V., Molineri A.I. (2023). Antimicrobial resistance of Streptococcus uberis isolated from bovine mastitis: Systematic review and meta-analysis. Res. Vet. Sci..

[22] Liang Z., Shen J., Liu J., Li Q., Yang F., Ding X. (2022). Ascorbic Acid-Mediated Modulation of Antibiotic Susceptibility of Major Bovine Mastitis Pathogens. Infect. Drug Resist..

[23] Martins L., Gonçalves J.L., Leite R.F., Tomazi T., Rall V.L.M., Santos M.V. (2021). Association between antimicrobial use and antimicrobial resistance of Streptococcus uberis causing clinical mastitis. J. Dairy Sci..

[24] Xu S., Liu Y., Gao J., Zhou M., Yang J., He F., Kastelic J.P., Deng Z., Han B. (2021). Comparative Genomic Analysis of Streptococcus dysgalactiae subspecies dysgalactiae Isolated From Bovine Mastitis in China. Front. Microbiol..

[25] Haenni M., Lupo A., Madec J.Y. (2018). Antimicrobial Resistance in *Streptococcus* spp.. Microbiol. Spectr..

[26] Rohmeier L., Jander S., Koy M., Macías L., Meyerholz M.M., Engelmann S., Hoedemaker M., Kuehn C., Schuberth H.-J., Seyfert H.M. (2017). Divergent genotype in Holstein heifers influences initial Staphylococcus aureus shedding after experimentally induced mastitis. Reprod. Domest. Anim..

[27] Giovannini A.E.J., van den Borne B.H.P., Wall S.K., Wellnitz O., Bruckmaier R.M., Spadavecchia C. (2017). Experimentally induced subclinical mastitis: Are lipopolysaccharide and lipoteichoic acid eliciting similar pain responses?. Acta Vet. Scand..

[28] Boireau C., Cazeau G., Jarrige N., Calavas D., Madec J.Y., Leblond A., Haenni M., Gay É. (2018). Antimicrobial resistance in bacteria isolated from mastitis in dairy cattle in France, 2006–2016. J. Dairy Sci..

[29] Xiao X., Chen X., Yan K., Jiang L., Li R., Liu Y., Wang M., Wang Z. (2022). PK/PD integration and pharmacodynamic cutoff of cefquinome against cow mastitis due to *Escherichia coli*. J. Vet. Pharmacol. Ther..

[30] Fu S., Wen C., Wang Z., Qiu Y., Zhang Y., Zuo J., Xu Y., Han X., Luo Z., Chen W. (2022). Molecular Epidemiology and Antimicrobial Resistance of Outbreaks of Klebsiella pneumoniae Clinical Mastitis in Chinese Dairy Farms. Microbiol. Spectr..

[31] Saddam S., Khan M., Jamal M., Rehman S.U., Slama P., Horky P. (2023). Multidrug resistant Klebsiella Pneumoniae reservoir and their capsular resistance genes in cow farms of district Peshawar, Pakistan. PLoS ONE.

[32] Wu X., Liu J., Feng J., Shabbir M.A.B., Feng Y., Guo R., Zhou M., Hou S., Wang G., Hao H. (2022). Epidemiology, Environmental Risks, Virulence, and Resistance Determinants of Klebsiella pneumoniae From Dairy Cows in Hubei, China. Front. Microbiol..

[33] Cheng J., Zhou M., Nobrega D.B., Barkema H.W., Xu S., Li M., Kastelic J.P., Shi Y., Han B., Gao J. (2021). Genetic diversity and molecular epidemiology of outbreaks of Klebsiella pneumoniae mastitis on two large Chinese dairy farms. J. Dairy Sci..

[34] Tartor Y.H., Abd El-Aziz N.K., Gharieb R.M.A., El Damaty H.M., Enany S., Soliman E.A., Abdellatif S.S., Attia A.S.A., Bahnass M.M., El-Shazly Y.A. (2021). Whole-Genome Sequencing of Gram-Negative Bacteria Isolated From Bovine Mastitis and Raw Milk: The First Emergence of Colistin mcr-10 and Fosfomycin fosA5 Resistance Genes in Klebsiella pneumoniae in Middle East. Front. Microbiol..

[35] Alves J.S., de Moura Souza R., Lima Moreira J.P., Gonzalez A.G.M. (2024). Antimicrobial resistance of Enterobacteriaceae and *Staphylococcus* spp. isolated from raw cow’s milk from healthy, clinical and subclinical mastitis udders. Prev. Vet. Med..

[36] Eidaroos N.H., Algammal A.M., Mohamaden W.I., Alenzi A.M., Alghamdi S., Kabrah A., El-Mahallawy H.S., Eid H.M., Algwad A.A., Asfor S.A. (2025). Virulence traits, agr typing, multidrug resistance patterns, and biofilm ability of MDR Staphylococcus aureus recovered from clinical and subclinical mastitis in dairy cows. BMC Microbiol..

[37] Dhital B., Chuang S.T., Hsieh J.C., Hsieh M.H., Chiang H.I. (2023). Prevalence, Virulence, and Antimicrobial Resistance of Major Mastitis Pathogens Isolated from Taiwanese Dairy Farms. Antibiotics.

[38] Landin H., Mörk M.J., Larsson M., Waller K.P. (2015). Vaccination against Staphylococcus aureus mastitis in two Swedish dairy herds. Acta Vet. Scand..

[39] Wilson D.J., Mallard B.A., Burton J.L., Schukken Y.H., Grohn Y.T. (2009). Association of *Escherichia coli* J5-specific serum antibody responses with clinical mastitis outcome for J5 vaccinate and control dairy cattle. Clin. Vaccine Immunol..

[40] Schukken Y.H., Bronzo V., Locatelli C., Pollera C., Rota N., Casula A., Testa F., Scaccabarozzi L., March R., Zalduendo D. (2014). Efficacy of vaccination on Staphylococcus aureus and coagulase-negative staphylococci intramammary infection dynamics in 2 dairy herds. J. Dairy Sci..

[41] Freick M., Frank Y., Steinert K., Hamedy A., Passarge O., Sobiraj A. (2016). Mastitis vaccination using a commercial polyvalent vaccine or a herd-specific Staphylococcus aureus vaccine. Results of a controlled field trial on a dairy farm. Tierarztl. Prax. Ausg. G. Grosstiere Nutztiere.

[42] Klaas I.C., Zadoks R.N. (2018). An update on environmental mastitis: Challenging perceptions. Transbound. Emerg. Dis..

[43] Leitner G., Yadlin N., Lubashevsy E., Ezra E., Glickman A., Chaffer M., Winkler M., Saran A., Trainin Z. (2003). Development of a Staphylococcus aureus vaccine against mastitis in dairy cows. II. Field trial. Vet. Immunol. Immunopathol..

[44] Chang B.S., Moon J.S., Kang H.M., Kim Y.I., Lee H.K., Kim J.D., Lee B.S., Koo H.C., Park Y.H. (2008). Protective effects of recombinant staphylococcal enterotoxin type C mutant vaccine against experimental bovine infection by a strain of Staphylococcus aureus isolated from subclinical mastitis in dairy cattle. Vaccine.

[45] Bradley A.J., Breen J.E., Payne B., White V., Green M.J. (2015). An investigation of the efficacy of a polyvalent mastitis vaccine using different vaccination regimens under field conditions in the United Kingdom. J. Dairy Sci..

[46] Perruchot M.H., Gondret F., Robert F., Dupuis E., Quesnel H., Dessauge F. (2019). Effect of the flavonoid baicalin on the proliferative capacity of bovine mammary cells and their ability to regulate oxidative stress. PeerJ.

[47] Jia F., Ma W., Zhang X., Wang D., Zhou X. (2020). Matrine and baicalin inhibit apoptosis induced by Panton-Valentine leukocidin of Staphylococcus aureus in bovine mammary epithelial cells. J. Dairy Sci..

[48] Zhao Q.Y., Yuan F.W., Liang T., Liang X.C., Luo Y.R., Jiang M., Qing S.Z., Zhang W.M. (2018). Baicalin inhibits *Escherichia coli* isolates in bovine mastitic milk and reduces antimicrobial resistance. J. Dairy Sci..

[49] Güran M., Çakıral K., Teralı K., Kandemir T., Şanlıtürk G., Öcal M.M., Nagiyev T., Köksal F. (2023). Meropenem in combination with baicalein exhibits synergism against extensively drug resistant and pan-drug-resistant Acinetobacter baumannii clinical isolates in vitro. Pathog. Dis..

[50] Ning B., Shen J., Liu F., Zhang H., Jiang X. (2023). Baicalein Suppresses NLRP3 and AIM2 Inflammasome-Mediated Pyroptosis in Macrophages Infected by Mycobacterium tuberculosis via Induced Autophagy. Microbiol. Spectr..

[51] Lu H., Li X., Wang G., Wang C., Feng J., Lu W., Wang X., Chen H., Liu M., Tan C. (2021). Baicalein Ameliorates Streptococcus suis-Induced Infection In Vitro and In Vivo. Int. J. Mol. Sci..

[52] Mao Y., Liu P., Chen H., Wang Y., Li C., Wang Q. (2023). Baicalein Inhibits the Staphylococcus aureus Biofilm and the LuxS/AI-2 System in vitro. Infect. Drug Resist..

[53] Arbab S., Ullah H., Bano I., Li K., Ul Hassan I., Wang W., Qadeer A., Zhang J. (2022). Evaluation of in vitro antibacterial effect of essential oil and some herbal plant extract used against mastitis pathogens. Vet. Med. Sci..

[54] Feng S., Zhang Y., Fu S., Li Z., Zhang J., Xu Y., Han X., Miao J. (2023). Application of Chlorogenic acid as a substitute for antibiotics in Multidrug-resistant *Escherichia coli*-induced mastitis. Int. Immunopharmacol..

[55] Lu Y., Hu Y.L., Kong X.F., Wang D.Y. (2008). Selection of component drug in activating blood flow and removing blood stasis of Chinese herbal medicinal formula for dairy cow mastitis by hemorheological method. J. Ethnopharmacol..

[56] Lopes T.S., Fussieger C., Theodoro H., Silveira S., Pauletti G.F., Ely M.R., Lunge V.R., Streck A.F. (2023). Antimicrobial activity of essential oils against Staphylococcus aureus and Staphylococcus chromogenes isolated from bovine mastitis. Braz. J. Microbiol..

[57] Bacic G. (2016). Intramammary Propolis Formulation for Subclinical Mastitis Prevention and Treatment in Dairy Cows. J. Dairy Vet. Anim. Res..

[58] Wang K., Jin X.L., Shen X.G., Sun L.P., Wu L.M., Wei J.Q., Marcucci M.C., Hu F.L., Liu J.X. (2016). Effects of Chinese Propolis in Protecting Bovine Mammary Epithelial Cells against Mastitis Pathogens-Induced Cell Damage. Mediat. Inflamm..

[59] Gill J.J., Pacan J.C., Carson M.E., Leslie K.E., Griffiths M.W., Sabour P.M. (2006). Efficacy and pharmacokinetics of bacteriophage therapy in treatment of subclinical Staphylococcus aureus mastitis in lactating dairy cattle. Antimicrob. Agents Chemother..

[60] Kwiatek M., Parasion S., Mizak L., Gryko R., Bartoszcze M., Kocik J. (2012). Characterization of a bacteriophage, isolated from a cow with mastitis, that is lytic against Staphylococcus aureus strains. Arch. Virol..

[61] Dias R.S., Eller M.R., Duarte V.S., Pereira Â.L., Silva C.C., Mantovani H.C., Oliveira L.L., Silva Ede A., De Paula S.O. (2013). Use of phages against antibiotic-resistant Staphylococcus aureus isolated from bovine mastitis. J. Anim. Sci..

[62] Fenton M., Keary R., McAuliffe O., Ross R.P., O’Mahony J., Coffey A. (2013). Bacteriophage-Derived Peptidase CHAP(K) Eliminates and Prevents Staphylococcal Biofilms. Int. J. Microbiol..

[63] Liu H., Li H., Liang Y., Du X., Yang C., Yang L., Xie J., Zhao R., Tong Y., Qiu S. (2020). Phage-delivered sensitisation with subsequent antibiotic treatment reveals sustained effect against antimicrobial resistant bacteria. Theranostics.

[64] Fuller R. (1991). Probiotics in human medicine. Gut.

[65] Martín R., Langa S., Reviriego C., Jiménez E., Marín M.L., Olivares M., Boza J., Jiménez J., Fernández L., Xaus J. (2004). The commensal microflora of human milk: New perspectives for food bacteriotherapy and probiotics. Trends Food Sci. Technol..

[66] Kitching M., Mathur H., Flynn J., Byrne N., Dillon P., Sayers R., Rea M.C., Hill C., Ross R.P. (2019). A Live Bio-Therapeutic for Mastitis, Containing Lactococcus lactis DPC3147 with Comparable Efficacy to Antibiotic Treatment. Front. Microbiol..

[67] Soleimani N., Kermanshahi R., Yakhchali B., Sattari T. (2010). Antagonistic activity of probiotic lactobacilli against Staphylococcus aureus isolated from bovine mastitis. Afr. J. Microbiol. Res..

[68] Shandilya U.K., Sharma A., Mallikarjunappa S., Guo J., Mao Y., Meade K.G., Karrow N.A. (2021). CRISPR-Cas9-mediated knockout of TLR4 modulates Mycobacterium avium ssp. paratuberculosis cell lysate-induced inflammation in bovine mammary epithelial cells. J. Dairy Sci..

[69] Deb R., Chaudhary P., De S. (2023). CRISPR/cas9 cassette targeting *Escherichia coli* (bla)CTX-M specific gene of mastitis cow milk origin can alter the antibiotic resistant phenotype for cefotaxime. Anim. Biotechnol..

[70] Liu J., Wei X., Zhang Y., Ran Y., Qu B., Wang C., Zhao F., Zhang L. (2024). dCas9-guided demethylation of the AKT1 promoter improves milk protein synthesis in a bovine mastitis mammary gland epithelial model induced by using Staphylococcus aureus. Cell Biol. Int..

[71] Jeong Y.G., Park J.H., Khang D. (2024). Sonodynamic and Acoustically Responsive Nanodrug Delivery System: Cancer Application. Int. J. Nanomed..

[72] Feng W., Teng Y., Zhong Q., Zhang Y., Zhang J., Zhao P., Chen G., Wang C., Liang X.J., Ou C. (2023). Biomimetic Grapefruit-Derived Extracellular Vesicles for Safe and Targeted Delivery of Sodium Thiosulfate against Vascular Calcification. ACS Nano.

[73] Wierzbicki M., Kot M., Lange A., Kalińska A., Gołębiewski M., Jaworski S. (2024). Evaluation of the Antimicrobial, Cytotoxic, and Physical Properties of Selected Nano-Complexes in Bovine Udder Inflammatory Pathogen Control. Nanotechnol. Sci. Appl..

[74] Wang W., Song Y., Petrovski K., Eats P., Trott D.J., Wong H.S., Page S.W., Perry J., Garg S. (2015). Development of intramammary delivery systems containing lasalocid for the treatment of bovine mastitis: Impact of solubility improvement on safety, efficacy, and milk distribution in dairy cattle. Drug Des. Devel. Ther..

[75] Hashem A.E., Elmasry I.H., Lebda M.A., El-Karim D., Hagar M., Ebied S.K.M., Alotaibi B.S., Rizk N.I., Ghamry H.I., Shukry M. (2024). Characterization and antioxidant activity of nano-formulated berberine and cyperus rotundus extracts with anti-inflammatory effects in mastitis-induced rats. Sci. Rep..

[76] Yadav R.K., Tripathi C.B., Saraf S.A., Ansari M.N., Saeedan A.S., Aldosary S., Rajinikanth P.S., Kaithwas G. (2019). Alpha-linolenic acid based nano-suspension protect against lipopolysaccharides induced mastitis by inhibiting NFκBp65, HIF-1α, and mitochondria-mediated apoptotic pathway in albino Wistar rats. Toxicol. Appl. Pharmacol..

[77] Liu D., Yang F., Xiong F., Gu N. (2016). The Smart Drug Delivery System and Its Clinical Potential. Theranostics.

[78] Zangabad P.S., Mirkiani S., Shahsavari S., Masoudi B., Masroor M., Hamed H., Jafari Z., Taghipour Y.D., Hashemi H., Karimi M. (2018). Stimulus-responsive liposomes as smart nanoplatforms for drug delivery applications. Nanotechnol. Rev..

[79] Silverman J.A., Deitcher S.R. (2013). Marqibo^®^ (vincristine sulfate liposome injection) improves the pharmacokinetics and pharmacodynamics of vincristine. Cancer Chemother. Pharmacol..

[80] Yavlovich A., Singh A., Blumenthal R., Puri A. (2011). A novel class of photo-triggerable liposomes containing DPPC:DC(8,9)PC as vehicles for delivery of doxorubcin to cells. Biochim. Biophys. Acta.

[81] Shrestha B., Wang L., Brey E.M., Uribe G.R., Tang L. (2021). Smart Nanoparticles for Chemo-Based Combinational Therapy. Pharmaceutics.

[82] Ma T., Zhang P., Hou Y., Ning H., Wang Z., Huang J., Gao M. (2018). “Smart” Nanoprobes for Visualization of Tumor Microenvironments. Adv. Healthc. Mater..

[83] Nielsen C., Emanuelson U. (2013). Mastitis control in Swedish dairy herds. J. Dairy Sci..

[84] Young C.W., Eidman V.R., Reneau J.K. (1985). Animal health and management and their impact on economic efficiency. J. Dairy Sci..

[85] Smith K.L., Hogan J.S. (1993). Environmental mastitis. Vet. Clin. N. Am. Food Anim. Pract..

